# Cystatin C is associated with poor survival in amyotrophic lateral sclerosis patients

**DOI:** 10.3389/fnins.2023.1309568

**Published:** 2024-01-05

**Authors:** Qirui Jiang, Yuan Guo, Tianmi Yang, Shirong Li, Yanbing Hou, Junyu Lin, Yi Xiao, Ruwei Ou, Qianqian Wei, Huifang Shang

**Affiliations:** ^1^Department of Neurology, Laboratory of Neurodegenerative Disorders, West China Hospital, Sichuan University, Chengdu, Sichuan, China; ^2^Outpatient Department, West China School of Nursing, West China Hospital, Sichuan University, Chengdu, Sichuan, China; ^3^Department of Neurology, Guizhou Provincial People’s Hospital, Guiyang, Guizhou, China

**Keywords:** amyotrophic lateral sclerosis, cystatin C, prognosis, clinical, biomarker

## Abstract

**Background:**

Cystatin C (CysC) levels in amyotrophic lateral sclerosis (ALS) have been found changes, however, the associations between serum CysC levels and the progression and survival of ALS remain largely unknown.

**Methods:**

A total of 1,086 ALS patients and 1,026 sex-age matched healthy controls (HCs) were enrolled in this study. Serum CysC, other renal function, and metabolic parameters were measured. Correlation analysis and binary logistic regression were used to explore the factors related to serum CysC. Kaplan–Meier curve and Cox regression model were used for survival analysis.

**Results:**

CysC levels were significantly higher in ALS patients compared to HCs (0.94 vs. 0.85 mg/L, *p* < 0.001). Compared with ALS patients with lower CysC levels, those with higher CysC levels had an older age of onset, significantly lower ALSFRS-R scores (40.1 vs. 41.3, *p* < 0.001), a faster disease progression rate (0.75 vs. 0.67, *p* = 0.011), and lower frontal lobe function scores (15.8 vs. 16.1, *p* = 0.020). In the correlation analysis, CysC levels were significantly negatively correlated with ALSFRS-R scores (*r* = −0.16, *p* < 0.001). Additionally, ALS patients with higher CysC levels had significantly shorter survival time (40.0 vs. 51.8, *p* < 0.001) compared to patients with lower CysC levels. Higher CysC levels were associated with a higher risk of death in Cox analysis (HR: 1.204, 95% CI: 1.012–1.433). However, when treatment was included in the model, the result was no longer significant.

**Conclusion:**

CysC levels in ALS patients were higher compared to HCs. Higher CysC levels were associated with greater disease severity, faster progression rate and shorter survival, needing early intervention.

## Introduction

Amyotrophic lateral sclerosis (ALS) is a fatal motor neurodegenerative disease as pathologically characterized by the progressive degeneration of both upper and lower motor neurons in the brain and spinal cord (Brown and Al-Chalabi, 2017). The distribution of survival in ALS patients varies widely, ranging from several months to more than 10 years, with a median survival time of 3 to 5 years from onset (Yang et al., 2022). Several factors, including clinical and demographic features, as well as genetic factors, have been explored as determinants of survival in ALS patients (Chen et al., 2022; Goutman et al., 2022). Regarding biomarkers, the neurofilament light chain (NfL) is currently a sensitive and commonly used marker, and serum NfL has been used as a biomarker of early stage and disease progression to guide clinical trials for ALS (Benatar et al., 2018, 2023). However, NfL is rarely used in clinical practice because of its high technical requirements and high price. Therefore, it is necessary to find more biomarkers related to ALS disease progression and prognosis with more extensive clinical application and lower measurement cost, in order to guide early diagnosis and clinical trials.

Cystatin C (CysC) is an endogenous cysteine proteinase inhibitor belonging to the type-II cystatin superfamily that is expressed in various tissues and plays an important role in the nervous system repair after injury and disease (Abrahamson et al., 1986; Gauthier et al., 2011). Some studies have found the alterations in CysC levels in neurodegenerative disease, such as Alzheimer’s disease (AD), Parkinson’s disease (PD) and ALS (Cathcart et al., 2005; Wilson et al., 2010; Chen et al., 2015, 2021; Ren et al., 2015; Cui et al., 2020; Nair et al., 2020; Yang et al., 2021; Zhang et al., 2022). However, there were inconsistent findings for ALS. For example, some studies found that plasma CysC levels of ALS patients was significantly increased and CysC levels in cerebrospinal fluid (CSF) was significantly decreased compared with healthy controls (HCs; Wilson et al., 2010; Zhu et al., 2018); some studies found no significant difference in the serum CysC level between ALS patients and controls (Tetsuka et al., 2013; Ren et al., 2015). Additionally, our previous study found CysC may be correlated with a decline in the cognitive function of patients with AD and multiple system atrophy (MSA; Chen et al., 2021; Zhang et al., 2022), as well as with the severity in MSA patients (Ye et al., 2021). However, other studies showed that serum CysC level was related to the disease severity and site of onset (Ren et al., 2015), as well as survival in ALS patients (Zhu et al., 2023), in contrast with some studies with negative findings (Tetsuka et al., 2013).

Therefore, in the current study, we aimed to investigate the relationship between serum CysC levels in ALS patients and HCs. Additionally, we examined the association between serum CysC levels and other blood measurements. Furthermore, we explored the potential correlation between serum CysC levels and disease progression as well as survival in a large cohort of Chinese ALS patients.

## Methods

### Participants

**Figure 1:**
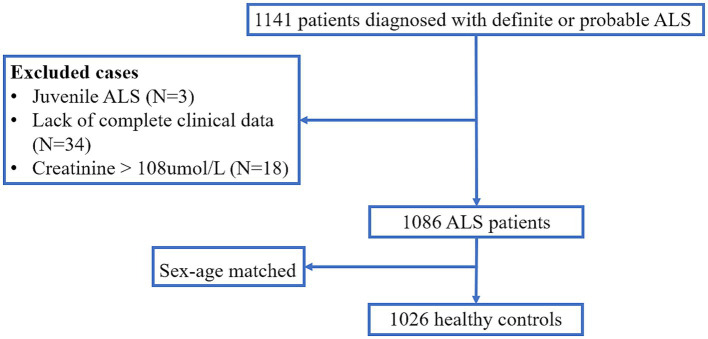
Flow chart the enrollment of cases.

The study was conducted in our tertiary referral center for motor neuron disease in southwest China (Department of Neurology, West China Hospital of Sichuan University, Chengdu, Sichuan province). A total of 1,141 patients diagnosed with definite or probable ALS according to the El Escorial revised criteria were included from August 2012 to June 2021 (Brooks et al., 2000). We excluded three juvenile ALS, 34 patients with incomplete data, and 18 patients with significantly elevated creatinine (creatinine >108 umol/L), and eventually included 1,086 ALS patients (Figure 1). To establish a comparison, we recruited 1,026 healthy controls (HCs) from the Health Management Center, West China Hospital of Sichuan University. HCs diagnosed with heart disease, hypertension, diabetes mellitus, or renal dysfunction, as well as any neurological diseases or psychiatric disorders were excluded. This study was approved by the Ethical Committee of West China Hospital of Sichuan University, and all ALS patients and control subjects signed written informed consent before participation.

### Data collection

Demographic data, including age, sex, education years, body mass index (BMI), and family history, as well as disease related data, including onset age, disease duration, diagnostic delay, onset region, and disease phenotypes, were collected at baseline. The ALS Functional Rating Scale revised (ALSFRS-R) scale was used to evaluate the functional impairment, and the rate of disease progression was evaluated by the changes of ALSFRS-R per month (Formula: (48-ALSFRS-R score at the baseline)/month intervals between first symptom onset and baseline).

All patients in the study underwent the Hamilton Depression Rating Scale-24 items (HDRS) to evaluate severity of depressive symptoms (Hamilton, 1960); Hamilton Anxiety Rating Scale (HARS) to evaluate severity of anxious symptoms (Hamilton, 1959). A HDRS score > 20 indicated the presence of depression and a HARS score > 14 indicated the presence of anxiety. The frontal lobe executive function was assessed by frontal assessment battery (FAB) in a face-to-face interview (Dubois et al., 2000). The Chinese version of Addenbrooke’s Cognitive Examination-revised (ACE-R) was adopted to evaluate global cognitive function in ALS patients. According to our previous studies, FAB score less than 16 was defined as frontal lobe dysfunction (Wei et al., 2014), and the ACE-R score less than 75 was defined as cognitive dysfunction according to our previous study (Wei et al., 2015).

All patients included were followed up by neurologists through telephone or in face-to-face interviews at 3- or 6-month intervals. All clinical and treatment data, including ALSFRS-R score, medication, and supportive treatment were collected during follow-up. Survival time was defined as the time interval from disease onset to the date of death for patients who passed away during the study or from disease onset to the last follow-up time for patients who were still alive at the end of the study.

### Blood parameters measurement

Samples were collected by venipuncture, performed between 8:00 and 11:00 a.m., after fasting from midnight. The blood specimens were left at room temperature for 30 min to clot and centrifuged for 10 min at 1,200 g. The CysC levels were measured by the automated particle-enhanced immunoturbidimetric method, and the measurement were completed in the Olympus AU5400 analyzer (Olympus, Tokyo, Japan) using the manufacturer’s reagents. In addition, other parameters of renal function (estimated glomerular filtration rate (eGFR), creatinine, uric acid, urea), nutrition (albumin) and glucose metabolism (glucose, HbA1c) and lipid metabolism (triglyceride, cholesterol, high-density lipoprotein, low-density lipoprotein) were also measured. All the experiments in the study were conducted in the Department of Laboratory Medicine, West China Hospital, Sichuan University.

### Statistical analysis

All analyses were performed using SPSS 26.0 (SPSS, Inc., Chicago, IL, United States). Continuous parameters that were normally distributed were described as the means ± standard deviation (SD), and those with a non-normal distribution were presented as the median (interquartile). Categorical variables were presented as percentages. Continuous variables were compared between the two groups using Student’s test or Mann–Whitney U test, and categorical variables were compared using Chi-square test. Spearman rank correlation coefficient was used to evaluate the correlation between baseline CysC levels and other hematological parameters. Partial correlation analysis was used to analyze the correlation between CysC levels and other hematological parameters. Binary logistic regression analysis was used to explore the potential factors associated with cognitive decline in ALS patients. The Kaplan–Meier (KM) curves and log-rank tests were used to assess survival in univariate analysis. Multivariable analysis was performed by Cox proportional hazards regression model to assess the effects of several variables on survival. The hazard ratios (HRs) and their 95% CIs were calculated. Two-tailed *p* < 0.05 was considered statistically significant.

## Results

The demographic features of the 1,086 ALS patients are shown in Table 1. The mean age of the patients was 54.3 ± 11.2 years, the mean disease duration was 15.4 ± 15.1 months, and the mean ALSFRS-R score was 40.7 ± 4.6. The CysC levels in ALS patients were increased significantly compared to age- and sex-matched HCs (0.94 vs. 0.85 mg/L, *p* < 0.001).

**Table 1:** Demographic and clinical information for ALS and healthy controls.

Variables	ALS patients	Healthy controls	*p* value
Number	1,086	1,026	NA
Age (y)	54.3 ± 11.2	54.4 ± 10.1	0.903
Sex (male, %)	62.1	60.6	0.472
Onset age (y)	53.1 ± 11.2	NA	NA
Onset region (bulbar, %)	15.3	NA	NA
Disease duration (m)	15.4 ± 15.1	NA	NA
ALSFRS-R score	40.7 ± 4.6	NA	NA
BMI (kg/m^2^)	22.7 ± 3.0	22.6 ± 2.1	0.358
Cystatin C level (mg/L)	0.94 ± 0.16	0.85 ± 0.12	<0.001*

ALS, amyotrophic lateral sclerosis; ALSFRS-R, ALS functional rating scale revised; BMI, body mass index.

According to the median (0.93 mg/L), the level of CysC of ALS patients was divided into two subgroups of higher or lower, and the demographic and clinical features of the two subgroups of ALS are shown in Table 2. In HCs, we also divided into two subgroups of CysC levels according to the median (0.84 mg/L), and the demographic of the two subgroups of HCs are shown in Table 2. We found that HCs with higher levels of CysC had an older age (57.9 vs. 50.7 years, *p* < 0.001) and a higher proportion of males (73.2% vs. 47.1%, *p* < 0.001) compared to those HC with lower levels of CysC. Additionally, we found a moderate positive correlation between CysC levels and age (*r* = 0.451, *p* < 0.001) and significantly higher levels in males compared to females (0.87 vs. 0.80 mg/L, *p* < 0.001). Similarly, compared with ALS patients with lower CysC levels, ALS patients with higher CysC levels had an older age of onset and a higher proportion of males. Additionally, patients with higher CysC levels had a significantly increased proportion of smoking and alcohol consumption (*p* < 0.001), significantly lower ALSFRS-R scores (40.1 vs. 41.3, *p* < 0.001), and a greater disease progression rate (0.75 vs. 0.67, *p* = 0.011) compared to those with lower CysC levels. There were no significant differences in the BMI, education, diagnostic delay, disease duration, proportion of early-stage patients, proportion of patients with different onset regions, HDRS score, HARS score, or FBI score (Table 2).

**Table 2:** Demographic and clinical characteristics of ALS patients and healthy controls with different levels of Cystatin C.

Variables	ALS patients			Healthy controls		
	Lower CysC levels *N* = 539	Higher CysC levels *N* = 547	*p* value	Lower CysC levels *N* = 493	Higher CysC levels *N* = 533	*p* value
Age (y)	50.9 ± 10.4	57.7 ± 11.0	<0.001^*^	50.7 ± 9.0	57.9 ± 9.9	<0.001^*^
Sex (male, %)	48.5	75.5	<0.001^*^	47.1	73.2	<0.001^*^
BMI (kg/m^2^)	22.74 ± 3.00	22.62 ± 3.02	0.515	NA	NA	NA
Onset age (y)	49.7 ± 10.4	56.4 ± 11.0	<0.001^*^	NA	NA	NA
Education (y)	9.3 ± 3.7	8.9 ± 3.7	0.184	NA	NA	NA
Smoking (never, %)	66.0	44.0	<0.001^*^	NA	NA	NA
Drinking (never, %)	70.3	57.5	<0.001^*^	NA	NA	NA
Diagnostic delay (m)	13.9 ± 13.9	14.6 ± 14.7	0.442	NA	NA	NA
Disease duration (m)	15.0 ± 14.3	15.7 ± 15.9	0.400	NA	NA	NA
Family history (%)	2.2	1.8	0.641	NA	NA	NA
Classical phenotype (%)	72.3	77.3	0.057	NA	NA	NA
Early stages (%)	73.4	71.7	0.562	NA	NA	NA
Onset region (bulbar, %)	17.3	13.3	0.074	NA	NA	NA
ALSFRS-R score	41.3 ± 4.3	40.1 ± 4.7	<0.001^*^	NA	NA	NA
ALSFRS-R bulbar	10.8 ± 1.7	10.8 ± 1.6	0.392	NA	NA	NA
ALSFRS-R limb	18.8 ± 3.9	17.5 ± 4.2	<0.001^*^	NA	NA	NA
ALSFRS-R respiratory	11.8 ± 0.6	11.7 ± 0.7	0.047^*^	NA	NA	NA
Disease progression rate	0.67 ± 0.63	0.75 ± 0.63	0.011^*^	NA	NA	NA
BMI at register	22.7 ± 3.0	22.6 ± 3.0	0.578	NA	NA	NA
HDRS score	9.1 ± 7.2	9.3 ± 7.3	0.656	NA	NA	NA
HARS score	6.4 ± 5.9	5.8 ± 5.7	0.074	NA	NA	NA
Anxiety (%)	9.6	6.9	0.107	NA	NA	NA
FAB	16.1 ± 2.1	15.8 ± 2.4	0.020^*^	NA	NA	NA
FAB<16 (%)	30.6	33.5	0.312	NA	NA	NA
FBI score	4.2 ± 6.3	3.7 ± 5.6	0.226	NA	NA	NA
ACE-R total score	79.3 ± 12.9	78.1 ± 13.7	0.253	NA	NA	NA
ACE-R < 75 (%)	30.2	33.8	0.206	NA	NA	NA

CysC, cystatin C; ALS, amyotrophic lateral sclerosis; ALSFRS-R, ALS functional rating scale revised; BMI, body mass index; HDRS, Hamilton Depression Rating Scale; HARS, Hamilton Anxiety Rating Scale; FAB, frontal assessment battery; eGFR, estimated glomerular filtration rate; FBI, frontal behavioral inventory; ACE-R, Addenbrooke’s cognitive examination-revised; a: Mean ± SE (95% CI). ^*^Significant difference.

Regarding cognition, FAB scores were significantly reduced in patients with higher CysC levels (15.8 vs. 16.1, *p* = 0.020), but there were no significant differences in ACE-R scores and the proportion of cognitive impairment (Table 2). However, in the binary logistic regression, we found that there was no significant correlation between CysC level and cognitive impairment (frontal executive function was assessed by FAB and overall cognitive function was assessed by ACE-R) in ALS patients (Supplementary Table S1).

**Figure 2:**
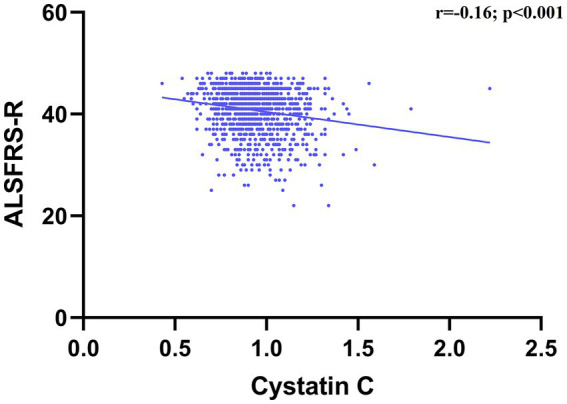
Association of cystatin C levels with disease severity.

**Figure 3:**
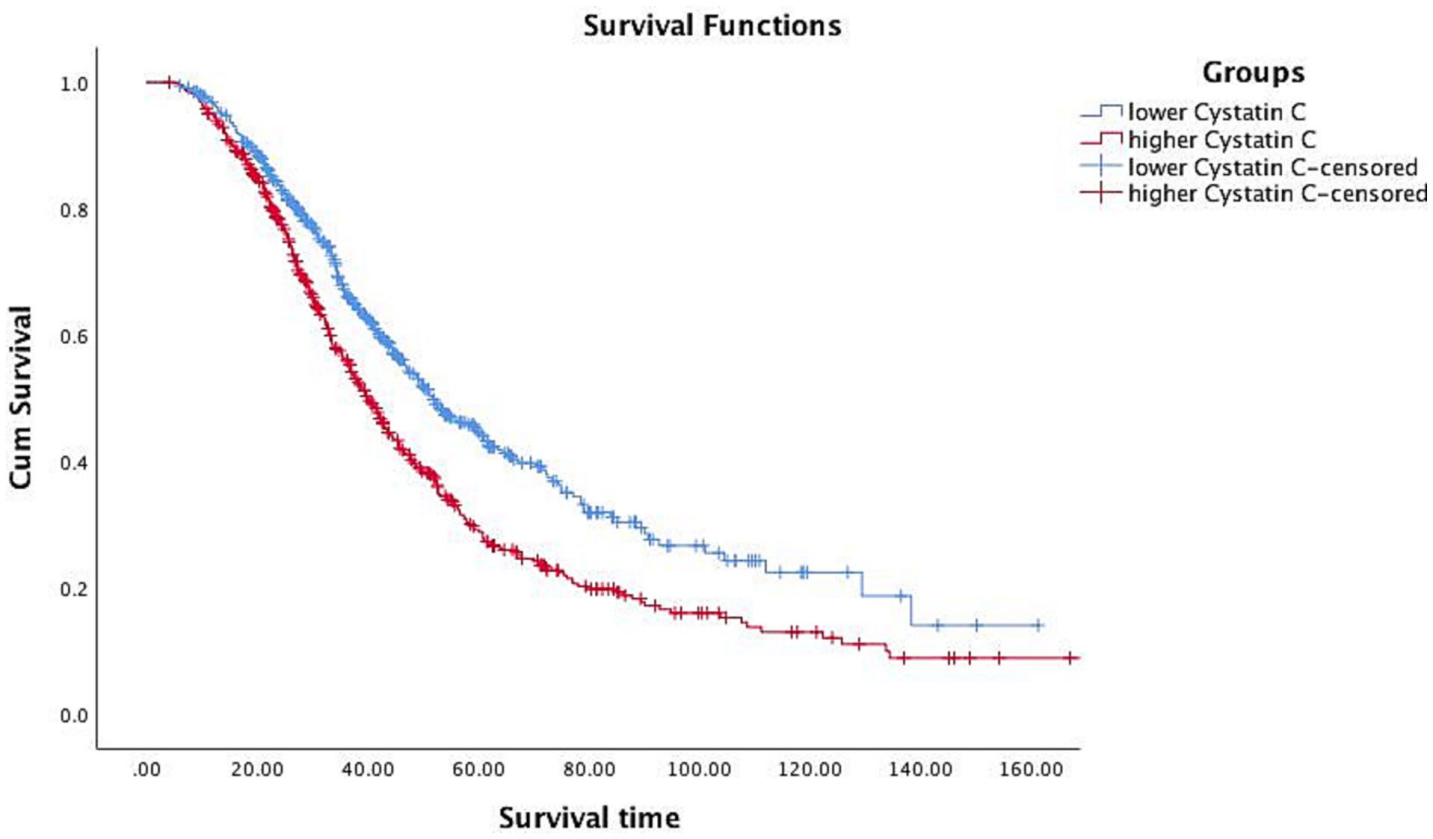
Survival analysis of patients with different cystatin C levels.

**Figure 4:**
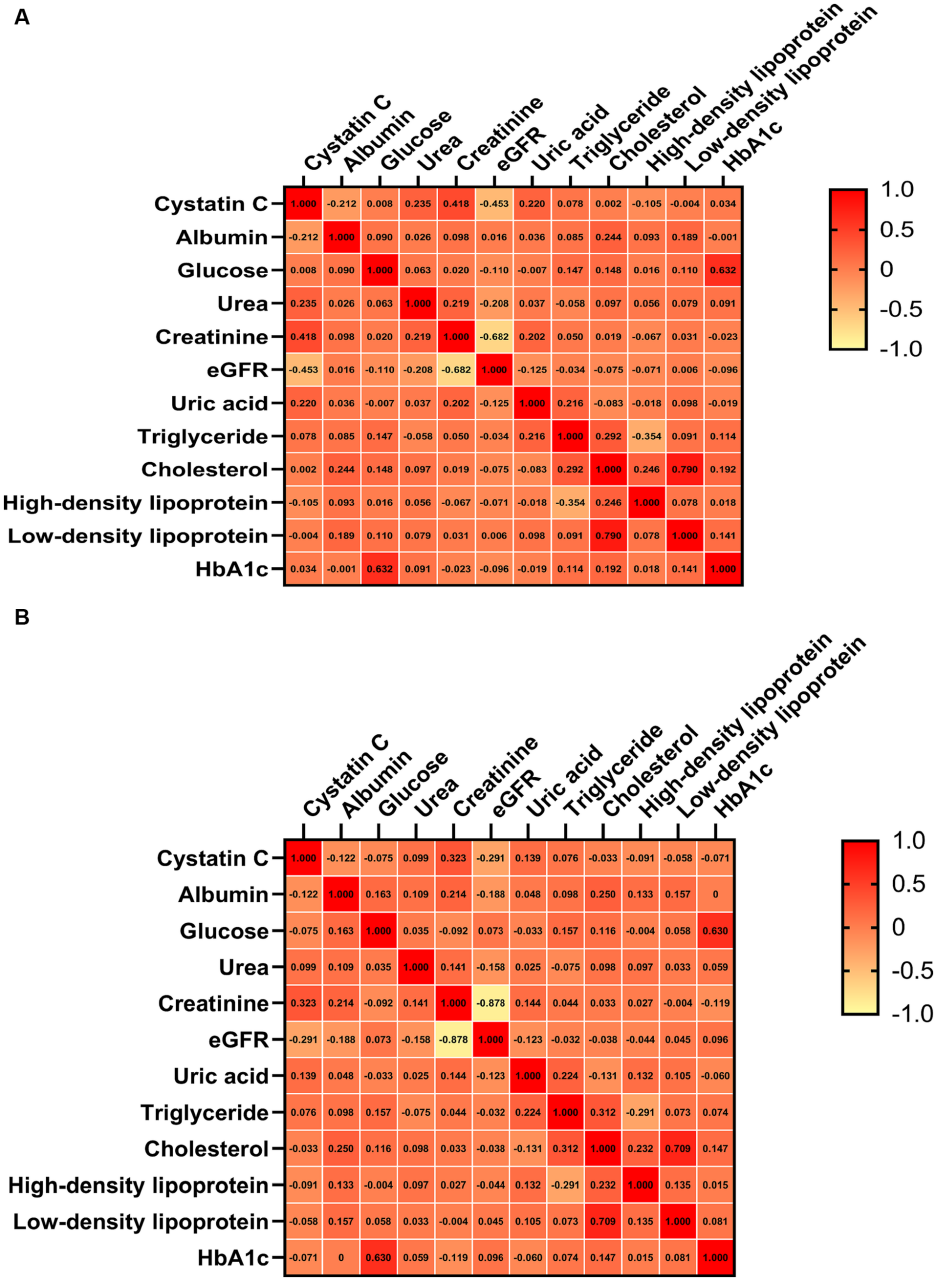
Correlation analysis of cystatin C level with other hematological parameters. **(A)** Unadjusted. **(B)** Adjusted sex, age, smoking (yes or no), drinking (yes or no) and BMI. eGFR, estimated glomerular filtration rate.

In the correlation analysis, after adjusting for sex, age, smoking (yes or no), drinking (yes or no) and BMI, we found that CysC levels was significantly negatively correlated with ALSFRS-R scores (*r* = −0.16, *p* < 0.001), although the correlation was relatively weak (Figure 2). Consistently, in terms of survival, we found that ALS patients with higher CysC levels had significantly shorter survival (40.0 vs. 51.8, *p* < 0.001; Figure 3). Furthermore, in the correlation analysis after adjusting for sex, age, smoking (yes or no), drinking (yes or no) and BMI, we found that CysC levels was significantly negatively correlated with albumin (*r* = −0.12, *p* = 0.004), eGFR (*r* = −0.29, *p* < 0.001), and positively correlated with creatinine (*r* = 0.32, *p* < 0.001), urea (*r* = 0.10, *p* = 0.020), uric acid (*r* = 0.14, *p* = 0.001). Although there was a significantly negative correlation between CysC level and high-density lipoprotein level, the coefficient was too weak to be considered relevant (*r* = −0.09, *p* = 0.033; Figure 4). Furthermore, we found ALS patients with higher CysC level had significantly higher levels of creatinine, urea, uric acid and triglyceride, and significantly lower eGFR and albumin compared to patients with lower CysC levels (Supplementary Table S2).

In the multivariate cox proportional hazard regression model, after adjusting for age, sex and ALSFRS-R score, higher levels of CysC were associated with a higher risk of death (HR: 1.204, 95% CI: 1.012–1.433; *p* = 0.036), indicating a 20.4% increase in mortality risk compared to lower CysC levels. However, after adjusting the treatment, the results were no longer significant (HR: 1.112, *p* = 0.234).

## Discussion

In our large ALS cohort, we analyzed the relationship between serum CysC level and clinical features, other hematological parameters, and prognosis of ALS. Firstly, we found that CysC level in ALS patients was significantly higher than that of matched HCs. Secondly, we found that CysC level was significantly negatively correlated with ALSFRS-R scores. Finally, we did not find the correlation between CysC levels and cognitive impairment in our cohort.

CysC is a 13.3-kDa basic protein produced by nucleated cells and widely expressed cysteine protease inhibitor with an abundance of about 13 times that of plasma in CSF (Barrett et al., 1984; George and Sheat, 1989). CysC is one of the proteins present in Bunina bodies (BB) inclusion bodies which is specific histological markers of ALS, and CysC immunoreactive BB is the characteristic of lower motor neurons (Okamoto et al., 1993; Yokota et al., 2006; Okamoto et al., 2008; Kimura et al., 2014). We found that serum CysC level of ALS patients was significantly higher than that of HCs. Some previous studies found that serum CysC level of ALS patients was not significantly different from that of HCs (Tetsuka et al., 2013; Ren et al., 2015), which was inconsistent with our results, possibly because of their small sample size of about 100 ALS patients. Our study found that the ALS patients with higher CysC levels had significantly older age of onset and higher proportion of male sex, which can be explained by that of there was a moderate positive correlation between CysC levels and age, and a significantly higher level in healthy males compared to healthy females. This finding was also supported by some previous studies (Ichihara et al., 2007; He et al., 2017; Ji et al., 2023). ALS patients with higher CysC levels had significantly high proportion of smoking, which was also supported by a healthy people study of finding that healthy smoking people had also significantly higher CysC level than that of non-smoking healthy people (Al Musaimi et al., 2019).

Creatinine, urea, eGFR, and uric acid are parameters of renal function. CysC is filtered in the glomeruli and fully reabsorbed and catabolic in the proximal tubules without being secreted (Laterza et al., 2002). Therefore, CysC is considered a potential substitute for serum creatinine estimates of GFR, and because CysC is less affected by muscle mass and diet than creatinine, previous studies generally expected that CysC would provide a more accurate estimate of GFR than creatinine (Tangri et al., 2011; Inker et al., 2012). However, in a large study it was found that estimates of GFR using equations based on CysC as the only filtration marker was no more accurate than creatinine-based estimate (Inker et al., 2012). In the current study, we did not include the ALS patients with abnormal GFR, therefore, higher level of CysC was not induced by renal dysfunction.

Our study found that serum CysC level in ALS was significantly negatively correlated with ALSFRS-R score and positively correlated with the of disease progression rate. One previous study found CysC levels in CSF of ALS patients was positively correlated with ALSFRS-R score and negatively correlated with disease progression rate (Ren et al., 2015). Another study also reported that ALS patients with lower CysC levels in CSF had shorter survival time compared to those with higher CysC levels in CSF (Wilson et al., 2010). A recent study also revealed similar findings (Zhu et al., 2023). Our study once again confirmed that patients with higher serum CysC level had faster disease progression and shorter survival than patients with lower CysC level. However, without adjusting the treatment, the other study demonstrated a significant positive correlation between CysC levels in CSF and survival time in ALS patients with limb onset (*r* = 0.486, *p* = 0.001), while no such correlation was observed in ALS patients with bulbar onset (Ryberg et al., 2010). Previous studies have also found differences in CysC levels between patients with upper limb and lower limb onset, and between limb onset and bulbar onset (Ren et al., 2015; Zhu et al., 2023). However, we did not find that CysC levels varied by site of onset in our ALS cohort, and although lower and lower CysC levels were associated with a higher proportion of bulbar onset, there was not statistically significant. This may be due to the smaller sample sizes of previous studies (92 ALS patients and 299 ALS patients). Therefore, combined with the results of our study, the relationship between CysC levels and the site of onset needs to be further explored. In mouse models, it was found that CysC has neuroprotective activity against ALS SOD-mediated toxicity, exogenous addition of CysC protects neuronal cells, including primary cultured motor neurons, depending on the coordinated activation of two different pathways: autophagy induction via AMPK-mTOR pathway and cathepsin B inhibition (Watanabe et al., 2014), the former can promote the degradation of misfolded or unfolded proteins and prevent the accumulation of abnormal mutant proteins, while the latter is closely related to the degeneration of motor neurons (Kikuchi et al., 2003). Subsequent studies have also shown that injection of CysC into the lateral ventricle can prolong the survival time in early symptomatic SOD1^G93A^ mouse (Watanabe et al., 2018), suggesting that CysC plays a neuroprotective role by counteracting the toxicity mediated by misfolded SOD1 protein (Watanabe et al., 2014, 2018). In our study, after adding treatment to the cox regression model, we found that higher CysC level was no longer associated with the risk of death compared to lower CysC levels. Therefore, more studies are necessary to clarify whether the level of CysC in CSF can better predict the death risk of ALS.

The study has some limitations. Firstly, we only measured CysC at a single point in time, but there was no long-term follow-up. Secondly, we did not adjust for other factors that could potentially impact the level of CysC, such as kidney disease, inflammation, tumors, and other underlying medical conditions. Finally, we only detected peripheral CysC, which may not fully reflect CysC level in the central nervous system, which would be better if combined with CysC in CSF.

## Conclusion

Our study, with the largest sample size to date, investigated the alterations in peripheral blood CysC levels in ALS patients. We observed a significant increase in serum CysC levels compared to HCs. Furthermore, higher serum CysC levels were associated with increased disease severity, faster progression rate, and shorter survival. However, further research is required to elucidate the underlying mechanisms by which CysC contributes to disease progression.

## Data availability statement

The raw data supporting the conclusions of this article will be made available by the authors, without undue reservation.

## Ethics statement

This study was approved by the Ethics Committee of West China Hospital of Sichuan University (approval no. 2015 (236)). The studies were conducted in accordance with the local legislation and institutional requirements. The participants provided their written informed consent to participate in this study.

## Author contributions

QJ: Conceptualization, Data curation, Methodology, Writing – original draft, Writing – review & editing. YG: Data curation, Supervision, Writing – original draft, Writing – review & editing. TY: Data curation, Supervision, Writing – review & editing. SL: Data curation, Writing – review & editing. YH: Data curation, Writing – review & editing. JL: Supervision, Writing – review & editing. YX: Supervision, Writing – review & editing. RO: Supervision, Writing – review & editing. QW: Conceptualization, Funding acquisition, Supervision, Writing – review & editing. HS: Conceptualization, Funding acquisition, Supervision, Writing – review & editing.
