# Supplementary material for: Cystatin C is associated with poor survival in amyotrophic lateral sclerosis patients
Source: Front Neurosci. 2024 Jan 5;17:1309568. doi: 10.3389/fnins.2023.1309568 (PMC10796561; doi:10.3389/fnins.2023.1309568)
Supplement: Supplementary file 1 [file Table_1.docx]

**Supplementary Table** **1.** **Factors associated with cognitive decline in ALS patients.**

| Variables | **FAB** | | | | | **ACE-R** | | | | |
| --- | --- | --- | --- | --- | --- | --- | --- | --- | --- | --- |
|  | B | SE | OR | 95% CI | P value | B | SE | OR | 95% CI | P value |
| Age (y) | -0.052 | 0.007 | 0.949 | 0.935-0.963 | <0.001* | 0.049 | 0.008 | 1.050 | 1.033-1.067 | <0.001* |
| Sex | -0.417 | 0.177 | 0.659 | 0.466-0.932 | 0.018* | 1.155 | 0.201 | 3.175 | 2.141-4.707 | <0.001* |
| Education (%) | 0.213 | 0.023 | 1.237 | 1.183-1.294 | <0.001* | -0.423 | 0.032 | 0.655 | 0.616-0.697 | <0.001* |
| Drinking (%) | -0.111 | 0.175 | 0.895 | 0.634-1.262 | 0.526 | 0.363 | 0.197 | 1.438 | 0.978-2.115 | 0.065 |
| Cystatin C | 0.138 | 0.158 | 1.148 | 0.842-1.565 | 0.384 | 0.176 | 0.176 | 1.193 | 0.845-1.685 | 0.317 |

Abbreviations: OR: odds ratios; CIs: confidence intervals. FAB: frontal assessment battery; ACE-R: addenbrooke’s cognitive examination-revised.

*Significant difference.

**Supplementary Table 2. Hematological parameters of ALS patients with different levels of Cystatin C.**

| Variables | Lower CysC levels  N=539 | Higher CysC levels  N=547 | P value |
| --- | --- | --- | --- |
| Albumin | 44.3 ± 3.6 | 42.9 ± 3.6 | <0.001* |
| Glucose | 5.3 ± 1.4 | 5.4 ± 1.7 | 0.466 |
| Creatinine | 56.6 ± 12.8 | 64.7 ± 13.5 | <0.001* |
| Urea | 5.1 ± 1.5 | 5.8 ± 1.6 | <0.001* |
| eGFR | 108.6 ± 13.4 | 99.0 ± 15.9 | <0.001* |
| Uric acid | 285.9 ± 68.4 | 343.7 ± 201.9 | <0.001* |
| Triglyceride | 1.4 ± 1.1 | 1.6 ± 1.0 | 0.023 |
| Cholesterol | 4.8 ± 1.0 | 4.7 ± 0.9 | 0.148 |
| High-density lipoprotein | 1.4 ± 0.4 | 1.4 ± 0.4 | 0.960 |
| Low-density lipoprotein | 2.9 ± 0.8 | 2.8 ± 0.9 | 0.453 |
| HbA1c | 5.7 ± 1.7 | 5.7 ± 1.7 | 0.665 |

eGFR: estimated glomerular filtration rate.
